# The Role of Tissue Macrophage-Mediated Inflammation on NAFLD Pathogenesis and Its Clinical Implications

**DOI:** 10.1155/2017/8162421

**Published:** 2017-01-01

**Authors:** Anna Alisi, Guido Carpino, Felipe L. Oliveira, Nadia Panera, Valerio Nobili, Eugenio Gaudio

**Affiliations:** ^1^Liver Research Unit of Bambino Gesù Children's Hospital and IRCCS, Rome, Italy; ^2^Department of Movement, Human and Health Sciences, Division of Health Sciences, University of Rome “Foro Italico”, Rome, Italy; ^3^Federal University of Rio de Janeiro, Institute of Biomedical Sciences, Rio de Janeiro, Brazil; ^4^Hepato-Metabolic Disease Unit of Bambino Gesù Children's Hospital and IRCCS, Rome, Italy; ^5^Department of Anatomical, Histological, Forensic Medicine and Orthopedics Sciences, Sapienza University of Rome, Rome, Italy

## Abstract

The obese phenotype is characterized by a state of chronic low-grade systemic inflammation that contributes to the development of comorbidities, including nonalcoholic fatty liver disease (NAFLD). In fact, NAFLD is often associated with adipocyte enlargement and consequent macrophage recruitment and inflammation. Macrophage polarization is often associated with the proinflammatory state in adipose tissue. In particular, an increase of M1 macrophages number or of M1/M2 ratio triggers the production and secretion of various proinflammatory signals (i.e., adipocytokines). Next, these inflammatory factors may reach the liver leading to local M1/M2 macrophage polarization and consequent onset of the histological damage characteristic of NAFLD. Thus, the role of macrophage polarization and inflammatory signals appears to be central for pathogenesis and progression of NAFLD, even if the heterogeneity of macrophages and molecular mechanisms that govern their phenotype switch remain incompletely understood. In this review, we discuss the role of adipose and liver tissue macrophage-mediated inflammation in experimental and human NAFLD. This focus is relevant because it may help researchers that approach clinical and experimental studies on this disease advancing the knowledge of mechanisms that could be targeted in order to revert NAFLD-related fibrosis.

## 1. Introduction

Nonalcoholic fatty liver disease (NAFLD) comprises more than one hepatic spectrum. It is a benign condition characterized by simple intrahepatic triglyceride accumulation (i.e., steatosis), which in turn may progress to a more severe form exhibiting steatosis, hepatocellular damage (i.e., ballooning), and tissue inflammation, collectively known as nonalcoholic steatohepatitis (NASH) [1]. However, this old paradigm has been challenged by several studies suggesting that patients with simple steatosis might not constitute a homogenous population. While some of these patients may progress unequivocally towards NASH, others may develop fibrosis directly, bypassing NASH as intermediate step [2].

Currently considered the most common liver disease worldwide, NAFLD is characterized by a rising prevalence in all age groups [3, 4]. It is widely accepted that the increased prevalence of NAFLD is strongly associated with the increasing prevalence of obesity. Development and progression of NAFLD are the result of a complex interplay between different organs and cell types. Indeed, the expansion of visceral adipose tissue and gut-derived endotoxins are key factors in NAFLD and its progression to fibrosis [5–10].

**Figure 1:**
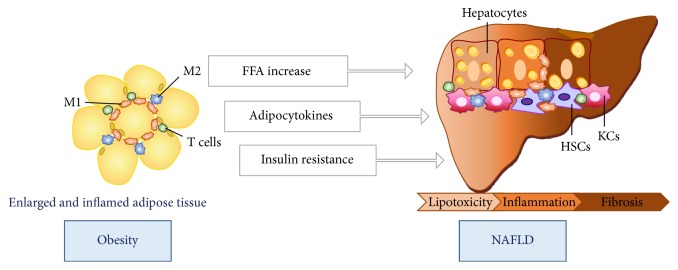
The crosstalk between adipose tissue macrophages and the liver cells in NAFLD.

During obesity, adipocyte size may dramatically increase. This hypertrophy promotes the rupture of adipocytes leading to a local inflammatory phenotype marked by the recruitment and activation of immune cells, such as macrophages and T cells, and by production of adipose tissue proinflammatory molecules (i.e., adipocytokines) that are released into circulation and can reach target organs, including the liver and skeletal muscle [5]. This aberrant activation of the immune response triggers harmful inflammation, which impairs the ability of insulin to inhibit free fatty acids (FFA) release causing their accumulation in the liver and consequent lipotoxicity, induces hepatic insulin resistance, and drives the low-grade inflammatory pattern of NASH and, later, liver fibrosis (Figure 1) [6].

Gut is now emerging as initiator of the events that contribute to obesity-associated systemic inflammation [7]. More specifically, obesity prone subjects exhibit alterations in gut microbiota balance (i.e., dysbiosis). A major compound of gut bacteria, lipopolysaccharide (LPS), plays a key role in hepatic inflammation and in macrophage polarization during NAFLD. Clinical evidence demonstrates that, in NAFLD subjects, LPS does not remain confined in the intestinal lumen but reaches the liver when the colonic mucosa immune function is impaired by gut dysbiosis [8, 9]. Toll-like receptor 4 (TLR-4) on the plasma membrane of liver-resident cells recognizes LPS as a ligand that prompts receptor dimerization and consequent activation of a signalling cascade. Next, the LPS/TLR-4 cascade causes the production of classical inflammatory cytokines, including tumour necrosis factor- (TNF-) *α*, interleukin- (IL-) 1*β*, and IL-6, exacerbating the hepatic inflammatory state and promoting fibrogenesis [10].

Despite what triggers the obesity-dependent proinflammatory response, advances in obesity research have led to the recognition of a primary role of the immune cells, such as macrophages and T-lymphocytes, in metabolic tissues. In particular, the crosstalk between tissue resident macrophages and adipocytes or hepatocytes appears to be for NAFLD development and progression. Therefore, macrophage-driven inflammation in NAFLD pathogenesis involves two different primary components that should be considered: one occurring in the adipose tissue and the other in the liver [11]. The identification of the pivotal molecules associated with the dynamic changes of macrophages and understanding their interactions could be crucial for designing novel therapeutic approaches against NAFLD.

Here, we review the role of macrophage-mediated inflammation in adipose tissue and the liver in NAFLD development and progression, highlighting the clinical implications of triggers and targets of macrophage activation towards a “maladaptive” phenotype.

## 2. Tissue Macrophage Polarization and Related Inflammation

Macrophages display high degrees of plasticity and heterogeneity. Functionally, macrophages can be divided into M1 to M2 subtypes that can be generated under different conditions [12, 13]. However, it is currently recognized that M1 and M2 phenotypes describe only extreme states towards which macrophages can be activated, because of several additional states that are distinct from both M1 and M2 or that simultaneously exhibit characteristics of M1 and M2 polarization [14, 15]. Even this implies that the pathogenesis of an inflammatory disease, such as NAFLD, could be characterized by a more complex pattern of shapes and functions that mark macrophages; we limited our literature review to the current characterization of M1 and M2 in general and NAFLD.

It has been reported that in vitro treatment with interferon- (IFN-) *γ*, TNF-*α*, and LPS induces M1 macrophage polarization. These M1 macrophages are considered proinflammatory or “classically activated” because they produce proinflammatory cytokines such as IL-1*β*, IL-6, IL-8, IL-12, and TNF-*α* and play a pivotal role in the triggering of tissue injury. By contrast, M2 macrophages differentiate in response to IL-4, IL-13, and IL-10 and are involved in tissue repair and efficient phagocytosis of cellular debris (efferocytosis). Therefore, they are considered tissue-repairing or “non-classically activated” macrophages [13]. Interestingly, wound healing is promoted by M2 macrophages through extracellular matrix (ECM) remodelling and recruitment of fibroblasts [16]. Furthermore, M2-secreted cytokines may support the function of T helper 2 cells. Finally, M2 macrophages may secrete a variety of matrix metalloproteases (MMPs), promoting the clearance of apoptotic cells and cellular debris [13]. This evidence highlights that M2 macrophages are versatile cells sharing several subtypes with different functions that appear to be dual. However, in physiological conditions, the main role of M2 macrophages is to create an anti-inflammatory milieu and promote tissue repair in the case of injury such as in the liver [17, 18]. During this process, the balance between TGF-*β*-dependent deposition of new ECM and its MMPs-mediated degradation promotes tissue repair but not pathological fibrosis. However, when the lesion persists M2 macrophages take an important profibrotic role and this cell population starts to secrete a very large amount of profibrotic factors such as TGF-*β* and galectin-3, as already reported in renal fibrosis [19].

**Figure 2:**
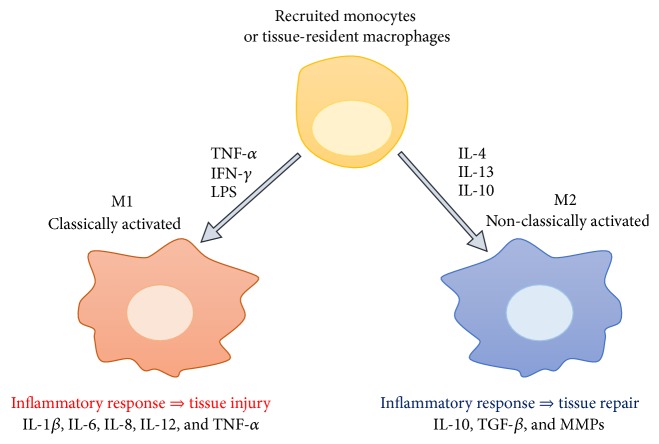
Schematic summary of macrophage polarization. Monocytes from peripheral blood differentiate in response to mediators in tissues, giving rise to different subsets: classically (M1) or alternatively (M2) activated macrophages that, respectively, lead to “bad” or “good” proinflammatory response.

The differentiation of tissue macrophages in M1 and M2 subsets is summarized in Figure 2.

In general, M1 and M2 macrophages exhibit different cell surface markers. M1 macrophages express high levels of CD80, CD86, CD68, and major histocompatibility complex class II [20], whereas M2 macrophages display an overexpression of other markers, including CD206, CD163, arginase-1 (Arg-1), dectin-1, scavenger receptors A and B-1, and C–C motif chemokine receptor-2 (CCR2) [15]. These and other markers of M1 and M2 macrophages are reported in Table 1.

Several inflammatory signals and transcription factors are involved in regulating macrophage polarization, including activation of the canonical interferon regulatory factor/signal transducer and activator of transcription (IRF/STAT) signalling, TLR-4/nuclear factor (NF)-*κ*B signalling, and transcription factors such as proliferator-activated receptors (PPARs) [21].

Understanding the above-mentioned mechanisms that drive monocyte recruitment, resident macrophage polarization, and the dissection of signalling pathways targeted by macrophage polarization may be critical to elucidate the precise role of adipose tissue macrophages (ATMs) and liver macrophage polarization during tissue necroinflammation in NASH. Therefore, in the next paragraphs we will discuss the general concepts and current evidence concerning inflammation and macrophage polarization in adipose and liver tissue in both human and experimental models of NAFLD.

## 3. Adipose Tissue Inflammation

### 3.1. General Characteristics

Adipose tissue is a complex immune organ composed of stromal-vascular cells (adipocytes and preadipocytes) and immune resident cells, including ATMs, T helper cells, cytotoxic T cells, B regulatory cells, and T regulatory cells, all of which play a role in maintaining immune balance [22].

In adipose tissue, M1 and M2 macrophages are phenotypically different. M1 macrophages express CD11b, CD11c, and F4/80 and secrete TNF-*α*, IL-1*β*, IL-6, nitric oxide (NO), and leukotriene B4, while M2 macrophages express CD11b, F4/80, CD301, and CD206 and produce anti-inflammatory cytokines such as IL-10 [23, 24]. Further markers for M1 and M2 ATMs are extensively reviewed by Hill et al. [25].

ATMs dispersed throughout lean adipose tissue have a predominant M2-like phenotype that helps to maintain local homeostasis [26]. Murine models of diet-induced obesity exhibit remodelling of the epididymal fat depot characterized by adipocyte death, ATM accumulation, and increase of depot weight [26]. In this context, the M1 phenotype appears to be primarily involved in tissue damage, proinflammatory cytokine secretion, leukocyte recruitment, and adipocyte expansion [27]. In obesity, high numbers of macrophages, mainly expressing M1 markers, accumulate in white adipose tissue (WAT) [28]. Over 90% of these macrophages surround dead adipocytes and form crown-like structures (CLS) [29]. Necrotic-like adipocytes are pathologic hallmarks of obesity and probably regulate macrophage homing in inflamed adipose tissue. In fact, necrotic-like adipocytes closed to multinucleate giant ATMs that secrete TNF-*α* and drive M1 macrophage response [28]. In contrast, apoptotic adipocytes induce M2 macrophage infiltration [29].

M1 accumulation macrophages in CLS are observed in fibrotic adipose tissue and M2 macrophages colocalization is associated with collagen VI, suggesting that M2 towards M1 polarization is a potential hallmark of inflammation/fibrosis in the adipose tissue [30].

It has been reported that macrophage-inducible C-type lectin (Mincle) is activated by endogenous ligands secreted by dead adipocytes and drives ATM migration in the course of CLS organization [31]. Mincle has previously been described as a proinflammatory marker of M1 polarization stimulated by TLR-4/nuclear factor- (NF-) *κ*B signalling, which is the main pathway involved in ATM activation [32, 33]. Another gene that may play a regulatory role during adipose tissue resident macrophage differentiation is Tribbles homolog 1 (Trib1). In fact, Trib-1-deficient mice exhibited more adipose tissue mass and fewer M2 macrophages [34].

Although the exact mechanism by which adipocytes control M1/M2 polarization in obese subjects is poorly known, it is evident that ATMs are crucial for immune-metabolism and their activation is associated with insulin resistance and consequent hepatometabolic effects, including NAFLD. The role of obesity-related inflammation in the development of insulin resistance was first suggested by experimental studies revealing that TNF-*α* increase/blocking was able to induce/decrease insulin resistance in in vitro and in vivo models [35–37]. However, during the last two decades, a significant advance in this field was recognition of the pivotal role of ATMs in the insulin resistance pathogenesis [38–40]. In particular, these series of studies showed the role of PPAR-*γ* in the switch of macrophage phenotype from M1 to M2 and its consequences on insulin resistance [38, 39]. Moreover, very recently, Lee et al. [41] demonstrated that epididymal natural killer cells have a critical role in controlling local ATM recruitment and adipose tissue inflammation, thereby regulating systemic insulin resistance in obesity.

### 3.2. Adipose Tissue Inflammation in Rodent Models of NAFLD

It has become increasingly evident that chemokines may play a key role in chronic subacute adipose tissue inflammation that is the common underlying condition of obesity, insulin resistance, and NAFLD [42]. Chemokines are small proteins that are expressed in different cells and tissues and control the trafficking of immune cells to sites of inflammation in a variety of conditions or diseases [43].

In an inflammatory condition, such as that occurring in obese WAT, chemokine (C-C motif) ligand (CCL2) binds its receptor CCR2 on a specific-subtype of circulating Ly6C^+^monocytes activating their transmigration and differentiation into M1 macrophages. While, in steady state, Ly6C^+^ monocytes differentiate into Ly6C^−^ monocytes that are prone to differentiate into M2 macrophages with an anti-inflammatory cytokine profile and involved in tissue repair [44, 45]. In high-fat diet (HFD) mice, which exhibit NAFLD features, adipose tissue is characterized by significant macrophage infiltration, inflammation, and tissue remodelling [46]. Moreover, HFD mice exhibit necrotic adipocytes that control Ly6C^+^ monocyte recruitment and subsequent differentiation into M1 macrophages [47]. Paradoxically, the CCR2 is classified as M2 macrophage marker in the current literature, but CCR2^+^ ATMs express prevalently M1 genes to the detriment of M2 genes during adipose tissue inflammatory response [47]. The overexpression of CCR2 by M1 macrophages in visceral WAT is associated with insulin resistance and consequently with NAFLD [48, 49]. Indeed, pharmacological antagonist of CCR2 reduced liver steatosis in obese and diabetic mice (db/db), and CCL2 deficiency reduced the accumulation of hepatic triglycerides in HFD and db/db mice [48, 50].

Another chemokine that may play a role in insulin resistance is CCL5. This chemokine has been found primarily involved in the migration of several immune cells by binding to its cognate receptors CCR1, CCR3, and CCR5 [51]. Kitade et al. [52] demonstrated that CCR5 regulated ATM recruitment and polarization and subsequent development of insulin resistance in WAT of genetically (ob/ob) and HFD obese mice.

In experimental NASH models, visceral adipose tissue of mice is enriched by clusters of CD11b^+^ macrophages producing IL-6 and TNF-*α* [53]. Consistently, the development of NASH in apolipoprotein E2 (APOE2) knock-in mice was attributed to activated CD68^+^ macrophages expressing proinflammatory genes in the liver [54]. ApoE regulates hepatic clearance of diet-derived chylomicrons and liver-derived low density lipoproteins remnants. In parallel, ApoE-deficient mice develop hyperlipidemia and atherosclerosis [55]. Moreover, M1 macrophage infiltration is frequently related to earlier events during spontaneous insulin resistance in mice [56].

In macrophage migration inhibitory factor- (Mif-) deficient obese mice, F4/80^+^Arg-1^+^IL-13^+^ M2 macrophages were predominant in the liver and strictly correlated with 70% reduction in F4/80^+^ ATMs and hepatoprotection [57]. Mif is a cytokine that may inhibit the migration of macrophages. In fact, Mif-deficiency did not affect obesity and lipid risk factors but did reduce inflammation in WAT and liver; it also reduced macrophage accumulation in WAT and blunted the expression of ICAM-1 and CD44 that regulate macrophage infiltration [58]. In this context, Mif is considered a potential therapeutic target for reducing the inflammatory component of metabolic and cardiovascular disorders. Consistently, hepatic triglycerides, type I collagen, and TGF-*β* mRNA expression as well as the size of adipocytes in visceral adipose tissue were substantially reduced after suppression of macrophage recruitment [59].

In summary, experimental models in rodents indicate that the kinetics of ATM mobilization seems to be important to establish an inflammatory response that shifts from adipose tissue to the liver, leading to NASH and other related metabolic diseases [60, 61].

### 3.3. Adipose Tissue Inflammation in Human NAFLD

The shift from M2 to M1 occurs also in adipose tissue of human obese subjects. Indeed, the genes encoding for CCL2, CCL8, CCL7, RANTES, CCL3, and CCL11 chemokines, as well as those encoding for CCR1, CCR2, CCR3, and CCR5, were found upregulated in the adipose tissue of morbidly obese compared with lean subjects [62]. Moreover, CD11c^+^ M1 macrophages expressing the inflammatory cytokines IL-6 and TNF-*α* increased in the adipose tissues of insulin resistant patients [28, 63].

Histological disturbances in the adipose tissue have been described with significant association between inflammation and ECM deposition. Obese patients with NASH showed high expression of tenascin-C by stromal-vascular fraction cells in a TNF-*α*-dependent manner [64]. Tenascin-C is a glycoprotein member of a damage associated molecular pattern rarely produced in healthy adipose tissue, but intensively synthesized during inflammation [65]. In human adipose tissue, tenascin-C is highly expressed by preadipocytes after macrophage stimulation by mechanisms involving LPS/TLR-4 signalling [66]. In parallel, TLR-4^−/−^ obese mice showed attenuated adipose tissue inflammation associated with preferential M2 macrophage polarization [67]. Based on these findings, it is plausible that tenascin-C deposition and LPS-dependent ATM polarization are critical to inflammation and ECM remodelling in visceral adipose tissue.

Recently, Du Plessis et al. [68] have analysed the transcriptional profile of subcutaneous and visceral adipose tissue of obese patients undergoing bariatric surgery. The authors found that the expression of proinflammatory genes was significantly increased in NAFLD and NASH patients in direct association with accumulation of CCR2^+^ M1 macrophages in visceral adipose tissue. These findings newly highlight that the role of CCR2 as a marker of a specific macrophage subtype is often controversial.

### 3.4. Possible Targets in ATM Inflammation

Among several potential targets that have been investigated as therapeutic applications in adipose tissue inflammation associated with NAFLD, chemokine/chemokine receptor system, adiponectin, leptin, and galectin-3 have attracted most attention due to their regulatory capacity on adipocyte and macrophage differentiation [42, 69–71].

As mentioned above, several lines of evidence demonstrated that CCR2 is crucial even if not exclusively responsible for ATM recruitment, thus suggesting the CCL2/CCR2 axis as a main target for therapy. Indeed, dampening ATM accumulation and consequent inflammation, via monocyte chemoattractant CCL2/CCR2 pharmacological inhibition, Tamura et al. [50] showed an improvement of obesity and related metabolic disorders, such as insulin resistance and hepatic steatosis in db/db mice. Moreover, recently it has been reported that macrophage-targeted delivery of small interference RNA against CCR2 inhibited ATM recruitment and accumulation in adipose tissue, thus reducing the downstream effects of obesity-induced inflammation [72].

Adiponectin has been described as an adipocyte-specific protein playing a positive role in the development of insulin resistance and atherosclerosis. It is negatively correlated with adiposity and its level is substantially reduced during obese-related inflammation [73]. Accordingly, adiponectin protein and mRNA levels are inversely correlated to TNF-*α* levels [74]. In humans, adiponectin induced M2 polarization and attenuated the expression of M1 markers by ATMs and stromal-vascular cells of adipose tissue [75]. Recently, it has been reported that macrophage polarization is crucial for the regulation of adiponectin receptor expression and differential adiponectin-mediated macrophage inflammatory responses [76]. Adiponectin reduces lipolysis in murine adipocytes [77]. The protective role of adiponectin was demonstrated in nSREBP-1c/adiponectin double-transgenic mice. The nSREBP-1c transgenic mice overexpress the nuclear sterol regulatory element-binding protein 1c (nSREBP-1c) in adipose tissue and develop hypoadiponectinemia and spontaneous liver disorders consistent with human NASH. The nSREBP-1c/adiponectin double-transgenic mice showed normal liver functions associated with the restoration of hepatic adiponectin production and circulating adiponectin levels [78]. Moreover, adiponectin-deficient mice exposed to HFD develop NASH-related fibrosis [79]. Previously, Nawrocki and colleagues demonstrated that adiponectin-deficient mice lost hepatic insulin sensitivity and response to PPAR-*γ*, indicating that adiponectin contributes to PPAR-*γ*-mediated improvements in glucose tolerance [80]. A recent metabolomic profiling of adiponectin-deficient mice indicated that lysophospholipid metabolism and *ω*-oxidation of fatty acids are directly regulated by adiponectin [81]. These findings suggest that adiponectin can be an anti-inflammatory protein with therapeutic potential to ameliorate symptoms of metabolic syndrome and NASH. However, it has been demonstrated that adiponectin should be used with caution because in M1 macrophages it may induce proinflammatory cytokines, whereas, in M2 macrophages, it may induce the anti-inflammatory cytokines [82].

Leptin is another important crucial adipokine involved in the pathogenesis of hepatometabolic effects of obesity. Indeed, it is known as a potent regulator of feeding behaviour and body weight, which has emerged by some seminal studies carried out in different mouse models of obesity [6, 83].

Noteworthily, the leptin receptor was found also on most immune cells including monocytes and macrophages. Moreover, as mentioned in previous paragraphs, ob/ob and db/db obese mice, respectively, deficient for leptin and leptin receptor, display a reduced ATM infiltration and inflammation [37]. Acedo et al. [71] showed that macrophages exposed to leptin treatment may promote a M2-like phenotype but induced proinflammatory cytokines release, such as TNF-*α* and IL-6. Luan et al. [84] demonstrated that the injection of leptin into ob/ob mice caused upregulation of circulating norepinephrine, increase of the cAMP content in epididymal fat pads, and HDAC4 dephosphorylation in WAT, triggering anti-inflammatory signals in ATMs.

Galectin-3 is a multifunctional *β*-galactoside binding protein firstly described on the macrophage surface [85] and widely associated with fibrosis in distinct tissues [86]. Galectin-3 interacts with distinct types of ECM glycoproteins, including tenascin-C [87]. As described above, tenascin-C is widely correlated with proinflammatory events during adipose tissue inflammation, but the interaction with galectin-3 during this process is poorly understood. In human adipose tissue, galectin-3 is synthesized predominantly by preadipocytes and activated macrophages [88]. Obese subjects are characterized by increased serum levels of galectin-3 that are directly correlated to growing body mass and age, as well as upregulation of circulating levels of leptin, resistin, and IL-6 [89].

In mice, recombinant galectin-3 induces preadipocyte proliferation [88]. Obese galectin-3 deficient mice are marked by increased visceral adipose tissue mass followed by accumulation of M1 macrophages. In contrast, in the same mice CD4^+^CD25^+^FoxP3^+^ T regulatory cell and M2 macrophage numbers decreased [90]. In macrophages, galectin-3 exhibits a high-affinity binding for advanced glycosylation end products (AGE), interfering with the pathogenesis of diabetic complications and other metabolic disorders [91]. Deficiency of galectin-3 corroborates with the pancreatic and renal damage associated with AGE accumulation and M1 polarization [92]. However, it is not clear if galectin-3 acts as a receptor for AGE (RAGE) in the course of adipose tissue inflammation and ATM differentiation.

The adipose tissue is able to modulate the expression of galectin-3 on macrophages. In vitro, preadipocytes inhibited the expression of galectin-3 by a specific subpopulation of macrophages, known as peritoneal-C2D macrophages, which retains plasticity in response to different microenvironments. Peritoneal-C2D macrophages that migrated to WAT expressed higher levels of galectin-3 than macrophages that moved to brown adipose tissue [93]. Moreover, in the course of monocyte-to-macrophage differentiation, galectin-3 mRNA and protein levels are substantially upregulated by M2 macrophages when compared with M1 macrophages [94].

Galectin-3 role on NAFLD pathogenesis has given mixed results [95, 96]. Recent studies highlight that galectin-3 targeting drugs may improve NAFLD-related liver damage, including intraportal and intralobular inflammatory tissue infiltrate, in mouse models [97]. Very recently, Li et al. [98] have demonstrated that galectin-3 knockout mice are protected from inflammation and insulin resistance. The authors showed that a small inhibitor of galectin-3 reduced insulin resistance in HFD mice by improving insulin sensitivity in myocytes and hepatocytes. This last study strongly supports the use of galectin-3 inhibitors as a new approach to treat obesity-related insulin resistance and its comorbidities.

However, to date, it is not clear if galectin-3 is crucial for M1/M2 polarization in both adipose tissue and liver during NAFLD and if the use of specific inhibitors against this protein may rescue M2/M1 ratio in these tissues.

## 4. Liver Tissue Inflammation

### 4.1. General Characteristics

Representing 80–90% of all tissue macrophages in the body, Kupffer cells (KCs) are located in the hepatic sinusoids and are central to innate immunity [99]. In normal conditions, this cell population can self-renew during adult life without the contribution of circulating monocytes [100]. Functionally, two subgroups of KCs can be recognized based on their phagocytic capabilities and cytokine production [101]. KCs in normal conditions exhibit an M2-like phenotype and express several receptors such as TLRs [102]. In the presence of TLR ligands, KCs become immunogenic and can induce T cell activation and the generation of an efficient cytotoxic T-lymphocytes response [102]. Furthermore, KCs are involved in the clearance of apoptotic cell debris and iron homeostasis via the expression of scavenger receptors. KCs can interact with multiple immune cells within the sinusoids, including T cells, dendritic cells, hepatic stellate cells (HSCs), and innate lymphocytes [102]. In inflammatory processes, KCs primarily drive the influx of inflammatory leukocytes such as neutrophils and monocytes.

Interestingly, KCs participate in the constitution of facultative stem cell niches in rodent and human liver; in adult liver, bipotential stem/progenitor cells (HPCs) are present and located in the finer branches of the biliary tree. HPCs minimally contribute to the normal turnover of liver parenchymal cells but are activated in the context of liver injuries [103]. The activation of HPCs is sustained by a specialized niche that furnishes several key signals driving HPC activity [104]. In normal conditions, HPCs are surrounded by endothelial cells, HSCs, and KCs, which release paracrine signals for the maintenance of the stem/progenitors in a quiescent state. In diseased livers, activated HSCs and inflammatory macrophages can produce distinct paracrine signals determining HPC activation and proliferation [104]. Moreover, in chronic liver diseases, macrophages are able to activate the canonical Wnt pathway in HPCs triggering their differentiation towards hepatocyte [105]. In particular, the efficient phagocytosis of the debris determines the secretion of WNT3a by KCs, thus activating the canonical Wnt pathway in nearby HPCs and triggering their differentiation towards hepatocyte [106].

Therefore, KCs are key orchestrators of cellular processes in healthy and injured liver. As discussed in the next paragraphs, several studies have indicated that KCs are central in numerous molecular and cellular frameworks and have a pivotal role in NAFLD-related inflammatory processes and fibrosis [106–116].

### 4.2. KCs and Liver Tissue Inflammation in NAFLD Animal Models

Experimental models have demonstrated that the activation of KCs represents a central event in the initiation and progression of liver injury [107, 108]. The central role of KCs in the pathogenesis of NAFLD has been suggested by several studies in mouse models where the ablation of KCs determined the marked reduction of hepatic insulin resistance and inflammation in diet-induced steatosis [109, 110]. In experimental NASH, macrophages are characterized by the accumulation of large amounts of toxic lipids [99, 111] and cholesterol crystals [112]; fat-laden KCs exhibit a switch to a proinflammatory (M1) phenotype, which is reversible by inhibition of lipogenesis [99, 113]. Moreover, data obtained by different research groups showed that chemical depletion of KCs was able to prevent the release of proinflammatory cytokines and to alleviate liver damage [114].

In the pathogenesis of NAFLD, the hepatic macrophage pool orchestrates several interactions and crosstalk among resident or recruited cells, thus driving inflammatory processes. In this context, several cellular signalling pathways trigger macrophage activation.

TLRs are able to induce KC activation towards the M1 phenotype; TLR-4 ablation determines the reduction of liver damage and the depletion of KCs in mice with NASH [115]. Similarly, leptin exerts proinflammatory effects triggering KC activation by a peroxynitrite-dependent mechanism [116]. Leptin can also stimulate inducible nitric oxide synthases (iNOS) and the resultant nitric oxide (NO) can react to produce peroxynitrite, a strong physiological oxidant, and can activate KCs towards a M1 phenotype [116]. In the context of NAFLD and metabolic syndrome, the conversion of arginine to NO and citrulline by NOS and its conversion to ornithine and urea by arginases have been of special interest. Induction of iNOS is a hallmark of M1 macrophages with the consequent production of oxidative stress [117]. Arg-1 is a key marker of M2 macrophages and confers anti-inflammatory properties by substrate competition with iNOS and through other mechanisms; M2 KCs can promote apoptosis of M1 KCs by an arginase-dependent mechanism, limiting liver injury and NASH progression [108]. Similarly, arginase-2 competes with iNOS for NO substrate and the balance between these two enzymes plays a crucial role in regulating immune responses and macrophage activation; arginase 2-knockout mice fed with a HFD showed profound changes in their livers, characterized by significant steatosis, inflammation, and marked M1 macrophage infiltration [118].

In general, signals leading to macrophage activation converge on two main downstream pathways, nuclear factor- (NF-) *κ*B and C-Jun N-terminal kinase (JNK) [119]. The JNK pathway is activated by reactive oxygen species, saturated free fatty acid, and cholesterol crystallization [119, 120]. Moreover, NF-*κ*B is a transcription factor that acts as a key regulator of inflammation and cell death and is activated by various stimuli, such as TLRs, IL-1*β*, and TNF-*α* [111]. Interestingly, in mice with NASH, hepatocytes with large lipid droplets and cholesterol crystals are surrounded by activated KCs aggregated in hepatic CLS [121]. The administration of cholesterol-lowering drugs causes the dissolution of cholesterol crystals and disperses CLS in obese mice [122]. All of these effects are associated with reduction of JNK activation and reversal of NASH [120].

Once activated towards the M1 phenotype, KCs secrete a variety of factors influencing inflammation and fibrosis. IL-1*β* is mainly produced by TLR-activated macrophages and has potent inflammatory effects; the lack of IL-1*α* or IL-1*β* inhibits transformation of steatosis to steatohepatitis and liver fibrosis in hypercholesterolemic ApoE-deficient mice [123]. Stienstra et al. [124] demonstrated that IL-1*β* was released by M1-polarized KCs and that this cytokine may promote triglyceride synthesis in hepatocytes by decreasing PPAR-*α* transactivating activity, which, in turn, inhibited fatty acid oxidation. Furthermore, selective deficiency of IL-1*α* in KCs reduces liver inflammation and expression of inflammatory cytokines [125]. In NASH, activated KCs also produce CCL2 that contributes to the recruitment of circulating monocytes and macrophages into the inflamed liver [7]. Indeed, choline-deficient amino acid-defined diet-induced steatosis, inflammatory cell infiltration, and liver fibrosis with increased hepatic expression of CCR2 and CCL2, while the KC depletion improved NASH with a decrease of CCL2 expression and recruitment of Ly6C^−^ monocytes that exhibit a typical M2 anti-inflammatory phenotype [126].

The importance of macrophage recruitment in NASH is further confirmed in macrophage migration inhibitory factor-knockout mice, which show higher fatty degeneration, liver inflammation, and macrophage recruitment [57].

Activated KCs can secrete TNF superfamily ligands such as TNF-*α* and TNF-related apoptosis-inducing ligand (TRAIL), thus inducing inflammation and apoptosis of adjacent hepatocytes [127]. The production of TNF-*α* by KCs contributes to hepatocyte apoptosis, increases monocyte recruitment, and is crucial for triggering NASH development [99]. Interestingly, the engulfment of KCs with apoptotic bodies can further stimulate the generation of ligands of the death receptor-signalling pathway, including TNF-*α* [128]. Experimentally, gadolinium chloride (a KC toxicant) attenuated the phagocytosis of apoptotic body and the production of ligands for death receptors by KCs; similar results were obtained by the inhibition of hepatocyte apoptosis [128]. This approach results in the attenuation of neutrophil infiltration and in the reduction of HSC activation, confirming the role of KCs and TNF-*α* in liver inflammation and fibrosis. Recently, the role of TRAIL signalling in obesity-associated inflammation has been further defined; genetic deletion of TRAIL receptor in obese mice suppressed NASH and reduced KC activation and accumulation of inflammatory macrophages in liver [129].

### 4.3. KCs and Liver Fibrosis: Molecular and Cellular Crosstalk in Murine Models

The spectrum of liver macrophage activation is also relevant for fibrosis progression in NAFLD. Recent studies have demonstrated the antifibrotic properties of KCs, which acting as M2 macrophages can produce a variety of MMPs, enhancing ECM degradation [130]. On the other side, M1 macrophages trigger fibrogenesis mainly by stimulating HSCs [111]. In normal conditions, HSCs are quiescent cells [131]. However, as a consequence of liver injuries, HSCs transdifferentiate into activated myofibroblast-like cells [132]. Activated HSCs begin to secrete ECM components and produce tissue inhibitors of metalloproteinases (TIMPs), thus altering the balance between ECM synthesis and degradation and leading to fibrosis [131].

Several molecular mechanisms form the basis for crosstalk between KCs and HSCs. M1 macrophages can activate HSCs by releasing TGF-*β* and other profibrogenetic cytokines, thus promoting collagen deposition and stimulating the production of TIMP-1 [133]. Moreover, KCs can promote HSC survival, inducing NF-*κ*B signalling via TNF-*α* and IL-1 secretion [131, 133]; furthermore, the secretion of several chemokines (i.e., CCL2, CCL3-5, CCL7 and CCL8) by macrophages can promote HSC migration [111]. As a consequence, KC depletion in mice models attenuates the progression of liver fibrosis [131].

On the other hand, KCs could be also implicated in promoting fibrosis resolution. In this context, specific subtypes of macrophages (M2) can secrete MMPs and TRAIL contributing to ECM degradation and HSC apoptosis, respectively [134].

Recent evidence indicates that HPC activation has a prominent role in the progression of liver fibrosis. Under pathological conditions, the activation of HPCs determines the appearance of the so-called ductular reaction (DR), which was recently found to be a main driver of liver fibrogenesis [131, 135]. In this context, the hepatic macrophage pool can influence the HPC response [131, 135]. Among the variety of macrophage cytokines, TNF-like weak inducer of apoptosis (TWEAK) has a key role in the expansion of undifferentiated HPCs [135]. Moreover, the capability of macrophages to remodel ECM influences the composition of the HPC niche and sustains HPC response and DR [105, 131, 135]. In turn, activated HPCs secrete a variety of substances such as TGF-*β*, Hedgehog (Hh) ligands, Osteopontin (OPN), and adipokines that are able to stimulate KCs and HSC, thus influencing inflammation and fibrogenesis [135].

### 4.4. Role of KCs in Liver Tissue Inflammation and Fibrosis in Human NAFLD

The role of KC activation in liver inflammation and fibrosis has been also elucidated in patients with NAFLD. Like adipose tissue in obesity, livers with NASH are characterized by the appearance of hepatic CLS [136]. These unique histological structures are correlated with hepatic inflammation and fibrosis [121]. Interestingly, in obese children with NAFLD, subcutaneous adipose tissue has CLS strictly correlated with liver fibrosis scores and diabetes risks [137].

The polarization of liver-resident macrophages is a key feature in NASH development. As previously indicated, CD163 is a surface scavenger receptor for haptoglobin-hemoglobin complexes expressed almost exclusively on M2 macrophages and monocytes. However, upon macrophage activation, CD163 is shed as its soluble form (sCD163) that can be measured in the circulation and serve as a biochemical marker of macrophage M1 activation [138]. sCD163 was associated with changes in NAFLD and metabolic profile during lifestyle intervention in obese children and in morbidly obese patients after bariatric surgery [139]. sCD163 increases in parallel with the severity of NAFLD and is reduced by lifestyle or surgical intervention, thus suggesting that macrophage M1 activation is reversible [139]. In this context, M2 KC polarization might protect against fatty liver disease morbidly injury [108].

Although NAFLD is conventionally assessed histologically for lobular features of inflammation, development of portal inflammation and fibrosis appears to be associated with disease progression in human patients [140]. The portal infiltrate is mostly constituted of macrophages and portal macrophage infiltration was the first change detected in patients with early NASH, even before elevated expression of proinflammatory cytokines. The presence of portal inflammation in NAFLD patients provides a link between macrophages and HPC activation. In both adult and paediatric patients, NASH development and fibrosis are associated with HPC activation and DR [131, 140, 141]. In parallel with animal studies, the portal macrophage infiltrate in human NAFLD may contribute directly to fibrogenesis as well as influence the fate of HPCs, regulating the balance between liver repair and fibrosis.

### 4.5. Possible Targets in Liver Tissue Inflammation

As discussed thus far, the recruitment of liver-resident macrophages (mainly KCs) and their polarization is a pivotal factor in obesity-associated insulin resistance and NAFLD/NASH. Therefore, even if there are not currently established therapies to revert NASH, several promising treatments targeting the hepatic activation and polarization of KCs in NASH are being developed [142].

The fact that KC recruitment and activation may be driven by chemokine/chemokine receptor system has prompted several experimental studies with pharmacological inhibitors of these pathways [136, 143]. In fact, Baeck et al. [136] found that mNOX-E36, which inhibited CCL2 by binding, reduced the amount of intrahepatic macrophages and proinflammatory cytokines and ameliorated hepatic steatosis in methionine-choline-deficient diet mice with NASH. Moreover, recently, cenicriviroc, a dual CCR2/CCR5 antagonist, was reported to be able to significantly reduce fibrosis and the NAFLD activity score in a NASH model [143]. On this basis, a phase 2 clinical trial addressing the effect of cenicriviroc in NASH patients with fibrosis is currently ongoing [144].

Macrophage activation can be influenced by the G protein-coupled receptor (GPR) 120. The protein GPR120 may modulate macrophage response by decreasing M1 proinflammatory and increasing M2 anti-inflammatory gene expression [108]. GPR120 exerts robust and broad anti-inflammatory effects, acting as a negative feedback signal on NF-*κ*B phosphorylation induced by TLRs and the TNF*α* cascade [145, 146]. Similarly, human KCs express GPR120 [147] and this expression in NAFLD patients can be modulated by the treatment with docosahexaenoic acid (DHA), the major dietary N-3 long-chain polyunsaturated fatty acid (LC-PUFA) [148–150]. Unfortunately, the effect of LC-PUFA alone seems to be restricted to early NAFLD stages. However, Carpino et al. [151] have recently reported that treatment with DHA determined a macrophage polarization towards a M2 phenotype in correlation with reduction of proinflammatory cytokines levels, increased macrophage apoptosis, and upregulation of macrophage Wnt3a expression in children with NASH.

Although the role of galectin-3 in NASH appears to be controversial, the antifibrotic effect of its absence is a certainty [95, 152]. Preliminary results of a randomized clinical trial with GR-MD-02, a galactoarabino-rhamnogalacturonan polysaccharide that is able to block the galectin-3 receptor, have been recently reported, thus supporting the planning of a phase 2 clinical trial in advanced fibrosis due to NASH [153].

Moreover, OPN represents an interesting molecular tool linking the crosstalk among KCs, HSCs, and HPCs. In the liver, OPN is produced by several cell types including T cells, macrophages, and HPCs [131, 154]. Upregulation of hepatic OPN was found in both humans and rodents with advanced NASH, while OPN-deficient mice were protected against NASH and fibrosis [131, 135, 154]. OPN may stimulate collagen synthesis in HSCs and exert an autocrine effect on HPCs [135, 154]. Furthermore, the ablation of OPN reduced HPC response, prevented fibrogenesis, and improved liver regeneration [155]. Interestingly, Kwon et al. [156] reported that Hh signalling can promote liver inflammation through OPN-mediated macrophage activation contributing to NAFLD progression, while the inhibition of Hh signalling can ameliorate hepatic inflammation in mice with NAFLD, highlighting the therapeutic propensity of Hh inhibitors [156].

## 5. Conclusion

ATM and hepatic macrophage polarization may be histological hallmarks of future preventive diagnoses, considering the earlier events on adipose tissue in comparison with those occurring in the liver of NAFLD patients. In order to confirm this hypothesis, further clinical studies on the expression of specific markers of M1 and M2 polarization are required. Several markers may differentiate M1 from the M2 subset, but to date only a few of them have been investigated in NAFLD and correlated with disease progression or with response to therapy. In this regard, it is also important to point out that functional heterogeneity of macrophages in NAFLD, such as in other diseases, is associated with a similar heterogeneity of the expression of specific markers that we overviewed here. A tissue array of the expression of M1/M2 populations in adipose tissue and liver could elucidate the dynamic changes of macrophage polarization and molecular networks orchestrating the switch of macrophage phenotype during NAFLD pathogenesis.

In addition, in both adipose tissue and liver tissue, it is necessary to investigate (i) the potential triggers that induce macrophage polarization towards a M1 “dark side” phenotype; (ii) the role of common molecular pathways; (iii) the link between triggers and liver necroinflammation and fibrosis; and (iv) the real role of M2 macrophages in NAFLD-related fibrogenesis. The dissection of these mechanisms could help in the identification of potential new therapeutic targets, improving the pharmacological therapy for pathophysiologic events of necroinflammation, ballooning, and fibrosis.

## Figures and Tables

**Table 1:** Typical surface markers and functions in M1 and M2 macrophages.

M1 markers	Major function	Ref.
CD80 (B7-1)	T cell activation and survival	[15]
CD86 (B7-2)	T cell activation and survival	[15]
MHC-II	Exogenous antigen presentation	[15]
CD11c	Phagocytosis	[17]
TLR4	Pathogen recognition	[18]
Mincle	Phagocytosis and proinflammatory cytokines	[18]

M2 markers	Major function	

CD206	Phagocytosis, antigen presentation, and resolution of inflammation	[19]
CD163	Tolerance induction and tissue regeneration	[15]
Dectin-1	Chemotaxis	[15]
CD301	Cell adhesion, cell-cell signalling, and glycoprotein turnover	[21]
Arginase-1	Suppression of clearance of intracellular pathogens	[15]

## Abbreviations

AGE: Advanced glycosylation end products
ApoE2: Apolipoprotein E2
Arg-1: Arginase-1
ATM: Adipose tissue macrophage
CCL: Chemokine (C-C motif) ligand
CCR: C–C motif chemokine receptor
CLS: Crown-like structures
DHA: Docosahexaenoic acid
DR: Ductular reaction
ECM: Extracellular matrix
GPR120: G protein-coupled receptor 120
HFD: High-fat diet
Hh: Hedgehog
HIF1: Hypoxia-inducible factor-1
HPCs: Hepatic progenitor cells
HSCs: Hepatic stellate cells
IFN-*γ*: Interferon-gamma
IL: Interleukin
iNOS: Inducible nitric oxide synthases
JNK: Jun N-terminal kinase
KCs: Kupffer cells
LC-PUFA: Long-chain polyunsaturated fatty acids
LPS: Lipopolysaccharide
MCP-1: Monocyte chemoattractant protein-1
MIF: Migration inhibitory factor
Mincle: Macrophage-inducible C-type lectin
MMPs: Matrix metalloproteases
NAFLD: Nonalcoholic fatty liver disease
NASH: Nonalcoholic steatohepatitis
NF-*κ*B: Nuclear factor-*κ*B
NO: Nitric oxide
nSREBP-1c: Nuclear sterol regulatory element-binding protein 1c
OPN: Osteopontin
PPARs: Peroxisome proliferator-activated receptors
RAGE: Receptor for AGE
TGF-*β*1: Transforming growth factor-beta 1
TIMPs: Tissue inhibitors of metalloproteinases
TLR: Toll-like receptor
TNF-*α*: Tumour necrosis factor-alpha
TRAIL: TNF-related apoptosis-inducing ligand
Trib1: Tribbles homolog 1
TWEAK: TNF-like weak inducer of apoptosis
WAT: White adipose tissue.
